# Prognostic performance of the NRS2002, NUTRIC, and modified NUTRIC to identify high nutritional risk in severe acute pancreatitis patients

**DOI:** 10.3389/fnut.2023.1101555

**Published:** 2023-03-02

**Authors:** Dayu Chen, Bing Zhao, Linyu Wang, Yusi Qiu, Enqiang Mao, Huiqiu Sheng, Feng Jing, Weihong Ge, Xiaolan Bian, Erzhen Chen, Juan He

**Affiliations:** ^1^Department of Pharmacy, Nanjing Drum Tower Hospital the Affiliated Hospital of Nanjing University Medical School, Nanjing, China; ^2^Department of Pharmacy, Ruijin Hospital Affiliated to Shanghai Jiao Tong University School of Medicine, Shanghai, China; ^3^Nanjing Medical Center for Clinical Pharmacy, Nanjing Drum Tower Hospital the Affiliated Hospital of Nanjing University Medical School, Nanjing, China; ^4^Emergency Department, Ruijin Hospital Affiliated to Shanghai Jiao Tong University School of Medicine, Shanghai, China; ^5^Department of Pharmacy, The Affiliated Cancer Hospital of Guangxi Medical University, Nanning, China; ^6^Department of Pharmacy, Guigang People’s Hospital, Guigang, China

**Keywords:** severe acute pancreatitis, NRS 2002, NUTRIC score, mNUTRIC score, nutritional risk, intensive care unit, mortality risk

## Abstract

**Background:**

Acute pancreatitis (AP) is the most common gastrointestinal disease requiring hospital admission. AP patients are categorized as mild, moderately severe, and severe AP (SAP). For SAP patients, malnutrition increases susceptibility to infection and mortality. The Nutritional Risk Screening 2002 (NRS 2002), the Nutrition Risk in Critically Ill (NUTRIC) score and modified Nutrition Risk in Critically Ill (mNUTRIC) are nutritional risk screening tools of critically ill patients and have not been validated in patients with SAP. It is essential to evaluate the prognostic performance of these nutritional risk screening tools.

**Materials and methods:**

A retrospective study was designed to validate the NRS 2002, NUTRIC, and mNUTRIC when applied to SAP patients. Receiver operating characteristic curves were plotted to investigate the predictive ability of clinical outcomes by comparing areas under the curve (AUC). Appropriate cut-offs were calculated by using Youden’s index. Patients were identified as being at high nutritional risk according to the calculated cut-off values. The effects of different scoring systems on mortalities were calculated using the Cox proportional hazards model. Logistic regression was used to assess the association between the energy provision and 28-day mortality.

**Results:**

From January 2013 to December 2019, 234 SAP patients were included and analyzed. Patients categorized as high nutritional risk by the NRS 2002 (12.6% versus 1.9% for 28-day and 20.5% versus 3.7% for 90-day), NUTRIC (16.2% versus 0.0% for 28-day and 27.0% versus 0.0% for 90-day), and mNUTRIC (16.4% versus 0.0% for 28-day and 26.4% versus 0.8% for 90-day) had significant higher mortality than those categorized as low nutritional risk. The NUTRIC (AUC: 0.861 for 28-day mortality and 0.871 for 90-day mortality, both cut-off value ≥3) and mNUTRIC (AUC: 0.838 for 28-day and 0.828 for 90-day mortality, both cut-off value ≥3) showed better predictive ability of the 28- and 90-day mortality than the NRS 2002 (AUC: 0.706 for 28-day mortality and 0.695 for 90-day mortality, both cut-off value ≥5).

**Conclusion:**

The NRS 2002, NUTRIC, and mNUTRIC scores were predictors for the 28- and 90-day mortalities. The NUTRIC and mNUTRIC showed better predictive ability compared with the NRS 2002 when applied to SAP patients.

## Introduction

According to the 2012 updated revision of Atlanta Classification of acute pancreatitis (AP) (1), mild AP is the most common form with no organ failure, local or systemic complications and usually can be resolved in the first week. Most patients with mild AP are self-limited, achieving full recovery in less than a week (2, 3). Unfortunately, unlike mild AP, moderately severe, and severe AP (SAP) have rather high mortality (4, 5). SAP is defined by persistent organ failure, that is, organ failure >48 h. In patients with SAP, the oral route is often not feasible, there is inadequate nutritional supplementation, and a protein deficiency will occur after the first week of hospitalization (6). Artificial nutrition is an important treatment in patients with SAP, and many patients with SAP have suffered worse outcomes due to inadequate nutritional supplementation. It has been established that this type of patient presents a marked inflammatory response, as well as one of the highest catabolic rates, regardless of the nutritional status before the onset of the disease (7). To such descriptions, in these patients there is a significant negative impact on the nutritional status and therefore should be considered a high nutritional risk. Therefore, early identification of patients at high nutritional risk and appropriate nutrition support is very important to improving outcomes resulting from the treatment of SAP and the patient’s quality of life (8).

The present ESPEN guidelines state that patients with SAP should be considered at high nutritional risk because of the catabolic nature of the disease and the significant impact of nutritional status on disease development (9). Scoring systems such as the Nutritional Risk Screening 2002 (NRS 2002) are recommended to identify patients at high nutritional risk, but this screening tool has not been validated for the specific population of patients with SAP.

The NRS 2002 was developed by Kondrup et al. two decades ago, and this nutritional risk assessment tool has since been used in patients with different diseases and been recommended by different guidelines (10–12). Nevertheless, there are no reports investigating and validating use of NRS 2002 in patients with SAP (8).

Heyland et al. previously proposed the Nutrition Risk in Critically Ill (NUTRIC) score, which is the first nutritional risk assessment tool developed and validated specifically for intensive care unit (ICU) patients and recommend by the American Society for Parenteral and Enteral Nutrition (ASPEN)/Society for Critical Care Medicine (SCCM) guidelines (12, 13). The score contains the variables of age, co-morbidities, days from hospital admission to ICU transfer, Acute Physiology and Chronic Health Evaluation II (APACHE II), Sequential Organ Failure Assessment (SOFA), and interleukin 6 (IL6). Applicability to routine clinical assessment was further expanded by waiving the requirement for determining IL6 in the modified NUTRIC score (mNUTRIC) (14). Many patients with SAP were admitted to the ICU because of systemic complications and failure of at least one organ. In that case, we considered whether both the NUTRIC and mNUTRIC scores, which were developed based on a population of critically ill patients, would be an option for a nutritional risk assessment tool for SAP patients. Unfortunately, neither the NUTRIC nor the mNUTRIC score have been validated in this population.

Thus, the purpose of this study was to investigate and potentially validate use of the NRS2002, NUTRIC score, and mNUTRIC score as nutritional risk assessment tools in SAP patients.

## Materials and methods

### Study design and patient enrollment

This was a retrospective study of patients suffering from SAP who were admitted to the ICU of Ruijin Hospital (China), a multidisciplinary unit in a university-affiliated tertiary care medical center, from January 2013 to December 2019. Adult patients (over 18 years of age) admitted to the ICU and diagnosed with SAP were included. SAP was diagnosed following the criteria of the Revised Atlanta Classification (1). The exclusion criteria were as follows: (1) ICU stay of less than 48 h; (2) abdominal surgery within 7 days before admission; (3) chronic pancreatitis; and (4) incomplete data. Given the nature of this retrospective observational study, no intervention, including nutritional practices, was made to standardize care. Enteral nutrition were managed to administered *via* nasojejunal tube within 72 h after admission. In cases of abdominal compartment syndrome and intolerance to enteral nutrition, supplement or total parenteral nutrition were started in not more than 10 days. The clinical protocols and management of patients was determined by the clinical team looking after the patient.

### Outcome measures and data collection

The primary outcomes were defined as all-cause mortality at 28 and 90 days. Secondary outcomes were use of a mechanical ventilator, renal replacement therapy, and vasoactive agents during the hospital stay; continuous (>48) use of a mechanical ventilator, renal replacement therapy, and vasoactive agents during the hospital stay; proportion of multiple-organ dysfunction syndrome (MODS); proportion of surgical intervention; and ICU length of stay. MODS was defined as the combined dysfunction of two major organ systems (1, 15).

Time from ICU admission to the start of nutrition therapy was recorded. The nutrition strategy at day 7 was also collected. If the patients died within 7 days after admission, the latest nutrition strategy was collected. The average calorie and protein intakes were calculated. The total calorie requirements were calculated as 25–30 kcal/kg/day and 1.2–1.5 g/kg/day protein as in the current guidelines (12, 16). Ideal body weight was used for obese patients with BMI > 25 kg/m^2^. The NRS 2002 was routinely performed and recorded at the time of ICU admission according to clinical practice. The CT severity index, Acute Physiology and Chronic Health Evaluation (APACHE II) scores, Sequential Organ Failure Assessment (SOFA) scores, NUTRIC scores, and mNUTRIC scores were calculated at the time of ICU admission. All clinical and laboratory parameters for the calculation of APACHE II, SOFA, NUTRIC, and mNUTRIC scores were recorded from the day of admission to ICU. Patients were followed up until death or observed for 90 days to conduct survival analyses 28 and 90 days after ICU admission.

### Statistical analysis

Continuous variables were tested for normal distribution using the Kolmogorov–Smirnov test and expressed as the mean ± standard deviation (SD) for the normally distributed data and as the median and quartiles (25th–75th) for skewed data distributions. Two-tailed Student’s *t*-test and Mann–Whitney test were used to analyze continuous data when appropriate. Categorical variables were presented as the number of cases. The Pearson chi-squared (*χ*^2^) test and Fisher’s exact test were used to analyze the categorical variables.

Receiver operating characteristic curves (ROC) were used to express the ability of different scoring systems for prediction of 28-day and 90-day mortalities *via* area under curve (AUC). Appropriate cut-offs were calculated by highest combined sensitivity and specificity using Youden’s index. Patients were identified as being at high nutritional risk according to the calculated cut-off values. Survival analyses were performed according to the Kaplan–Meier curves; all deaths were recorded as events. The log-rank (Mantel–Cox) test was used for the comparison of survival curves. Relationship between 28-day mortality and nutrition strategy in patients identified as high nutritional risk by different tools was also analyzed. The effects of different screening tools on mortalities were also calculated using the Cox proportional hazards model. The results are reported as the hazard ratio (HR) with 95% confidence interval (CI). Subgroup analyses, with Cox proportional hazards adjusted for the same covariates as in the main model, were conducted to assess the interactions between different characteristics. The following prespecified baseline characteristics were analyzed: sex (male versus female); age (>55 versus ≤55); APACHE II score (≥8 versus <8); white-cell counts (>16,000 versus ≤16,000/mm^3^); CT severity index (>6 versus ≤6); C-reactive protein level (>150 versus ≤150 mg/L); serum creatinine level (≥1.8 versus <1.8 mg/dl), and etiology (biliary versus non-biliary). Logistic regression was used to assess the strength of the association between the energy provision and 28-day mortality. Three logistic models including three different nutritional risk screening tools (the NRS 2002, NUTRIC score and mNUTRIC score), the energy provision and their product (interaction) were performed to assess if the nutritional risk screening tools modified the association between energy provision and 28-day mortality. Finally, the logistic models were run separately in patients categorized as low and high nutritional risk by three screening tools. The results are reported as the odds ratio (OR) with 95% CI. The statistical significance of lack of fit was tested by the Hosmere-Lemeshow goodness of fit test. The significance was assumed at a *p*-value <0.05. IBM SPSS Statistics software (version 25.0; Chicago, IL, United States) and GraphPad Prism 9.2 (GraphPad Software, La Jolla, CA, United States) were used for statistical analysis and plotting graphs.

## Results

### Patient characteristics

A total of 343 critically ill patients with SAP were initially included. Then, 49 patients were excluded for incomplete data, 26 were excluded for staying in the ICU less than 48 h, 24 were excluded for previous abdominal surgery, and 20 were excluded for chronic pancreatitis. A total of 234 patients were finally included in the analysis. The patient characteristics are presented in Table 1.

**Table 1:** Patient characteristics.

Variables	Overall population (*n* = 234)
Demographics	
Age (years)	47 (37–62)
Sex (male, %)	156 (66.7)
BMI (kg/m^2^)	23.7 ± 3.9
NRS 2002	5 (4–5)
NUTRIC	3 (2–4)
mNUTRIC	3 (2–4)
APACHE II	9 (5–13)
SOFA	4 (2–6)
CT severity index at admission	6 (4–7)
MAP at admission (mmHg)	100 (92–111)
Calories received within first week (kcal/kg/day)	15.4 ± 3.2
Etiology	
Biliary (*n*, %)	91 (38.9)
Alcoholic (*n*, %)	62 (26.5)
Hypertriglyceridemia (*n*, %)	62 (26.5)
Other (*n*, %)	19 (8.1)
Laboratory test	
PCT at admission (ng/ml)	1.1 (0.4–6.1)
CRP at admission (mg/L)	180 (91–249)
White-cell count at admission (/mm^3^)	13,020 (9590–17,320)
Serum creatinine at admission (mg/dl)	0.8 (0.6–1.3)
Serum amylase at admission (IU/L)	511 (197–1,163)
Clinical outcomes	
28-day mortality (*n*, %)	18 (7.7)
90-day mortality (*n*, %)	30 (12.8)
Length of ICU stay (days)	30 (18–44)
Surgical intervention (*n*, %)	25 (10.7)
Use of mechanical ventilator (*n*, %)	194 (82.9)
Renal replacement therapy (*n*, %)	83 (35.5)
Use of vasoactive agent (*n*, %)	36 (15.4)
Use of mechanical ventilator >48 h (*n*, %)	31 (13.3)
Renal replacement therapy >48 h (*n*, %)	55 (23.5)
Use of vasoactive agent >48 h (*n*, %)	22 (9.4)
MODS (*n*, %)	75 (32.1)

BMI, body mass index; NRS 2002, Nutritional Risk Screening 2002; NUTRIC, Nutrition Risk in Critically Ill; mNUTRIC, modified Nutrition Risk in Critically Ill; APACHE, Acute Physiology and Chronic Health Evaluation; SOFA, Sequential Organ Failure Assessment; PCT, procalcitonin; CRP, C-reactive protein; MAP, mean artery pressure; MODS, multiple-organ dysfunction syndrome.

### Clinical outcomes predicted by the NRS 2002, NUTRIC, and mNUTRIC

**Figure 1:**
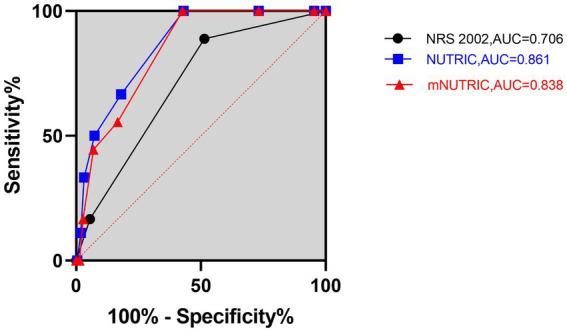
Prognostic accuracy of the NRS 2002, NUTRIC, and mNUTRIC to predict 28-day mortality analyzed by receiver operating characteristic curves. The black line with cycle represents the results of the NRS 2002 (AUC = 0.706, 95% CI: 0.595–0.817, *p* = 0.004). The blue line with square represents the results of the NUTRIC (AUC = 0.861, 95% CI: 0.794–0.929, *p* < 0.001). The red line with triangle represents the results of the mNUTRIC (AUC = 0.838, 95% CI: 0.768–0.908, *p* < 0.001).

**Figure 2:**
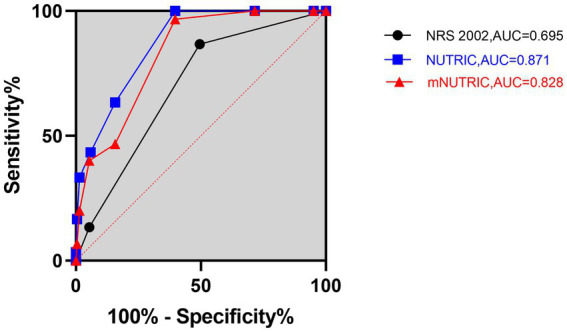
Prognostic accuracy of the NRS 2002, NUTRIC, and mNUTRIC to predict 90-day mortality analyzed by receiver operating characteristic curves. The black line with cycle represents the results of the NRS 2002 (AUC = 0.695, 95% CI: 0.604–0.787, *p* = 0.001). The blue line with square represents the results of the NUTRIC (AUC = 0.871, 95% CI: 0.818–0.925, *p* < 0.001). The red line with triangle represents the results of the mNUTRIC (AUC = 0.828, 95% CI: 0.754–0.891, *p* < 0.001).

The 28-day mortality was 7.7%, and the 90-day mortality was 12.8%. The mortality rates in SAP patients according to the NRS 2002, NUTRIC, and mNUTRIC are illustrated in Supplementary Figure S1. The predictive ability for 28-and 90-day mortality risk were analyzed by ROC, and the results are shown in Figures 1, 2. As depicted in Figures 1, 2, both the NUTRIC and mNUTRIC showed a reasonable ability to predict 28-and 90-day mortality in SAP patients. The NUTRIC and mNUTRIC performed better than the NRS 2002 in predicting both the primary and secondary outcomes.

The NUTRIC (AUC = 0.861, 95% CI: 0.794–0.929, *p* < 0.001) and mNUTRIC (AUC = 0.838, 95% CI: 0.768–0.908, *p* < 0.001) showed a higher predictive value than the NRS 2002 (AUC = 0.706, 95% CI: 0.595–0.817, *p* = 0.004), and thus better performance, in predicting 28-day mortality. In the prediction of 28-day mortality, the highest combined sensitivity and specificity of the NRS 2002 was found with a cut-off value of ≥5 (sensitivity = 88.9%, specificity = 52.1%). The NUTRIC had a cut-off value of ≥3 (sensitivity = 100%, specificity = 43.1%). The cut-off value of the mNUTRIC was also found at ≥3 (sensitivity = 100%, specificity = 42.6%). The NUTRIC (AUC = 0.871, 95% CI: 0.818–0.925, *p* < 0.001) and mNUTRIC (AUC = 0.828, 95% CI: 0.754–0.891, *p* < 0.001) also performed better than the NRS 2002 (AUC = 0.695, 95% CI: 0.604–0.787, *p* = 0.001) in predicting 90-day mortality. The cut-off values were the same in predicting the 28-day and 90-day mortality (≥5 for NRS 2002 and ≥3 for both NUTRIC and mNUTRIC). The results of the ROC analyses to predict the clinical outcomes are shown in Table 2.

**Table 2:** Prognostic accuracy of the NRS2002, NUTRIC, and mNUTRIC to predict clinical outcomes analyzed by ROC.

Clinical outcomes	NRS 2002			NUTRIC			mNUTRIC		
	AUC	95% CI	*p*-Value	AUC	95% CI	*p*-Value	AUC	95% CI	*p*-Value
28-day mortality	0.706	0.594–0.817	0.004	0.861	0.794–0.929	<0.001	0.838	0.768–0.908	<0.001
90-day mortality	0.695	0.604–0.787	0.001	0.871	0.818–0.925	<0.001	0.828	0.764–0.891	<0.001
Surgical intervention	0.661	0.561–0.761	0.009	0.727	0.637–0.817	<0.001	0.696	0.605–0.787	0.001
Use of mechanical ventilator	0.658	0.571–0.745	0.002	0.716	0.618–0.815	<0.001	0.717	0.619–0.815	<0.001
Renal replacement therapy	0.575	0.500–0.650	0.057	0.545	0.458–0.632	0.257	0.537	0.451–0.624	0.348
Use of vasoactive agent	0.678	0.585–0.771	0.001	0.730	0.622–0.839	<0.001	0.712	0.604–0.819	<0.001
Use of mechanical ventilator >48 h	0.663	0.565–0.760	0.004	0.698	0.609–0.787	<0.001	0.686	0.599–0.774	0.001
Renal replacement therapy >48 h	0.547	0.463–0.632	0.288	0.550	0.444–0.656	0.262	0.542	0.437–0.646	0.349
Use of vasoactive agent >48 h	0.664	0.552–0.776	0.011	0.709	0.584–0.834	0.001	0.683	0.559–0.806	0.005
MODS	0.702	0.633–0.771	<0.001	0.754	0.686–0.823	<0.001	0.741	0.672–0.810	<0.001

The NUTRIC and mNUTRIC had similar performance in predicting secondary outcomes. The NRS 2002 was the least valuable scoring system for predicting the clinical outcomes. All three scoring systems had no prognostic relevance with the use or continuous use (>48 h) of renal replacement therapy in patients with SAP. A comparison of the clinical outcomes in SAP patients categorized as high nutritional risk and low nutritional risk is shown in Table 3. Other characteristics are demonstrated in Supplementary Table S1.

**Table 3:** Comparison of clinical outcomes in SAP patients categorized as high nutritional risk and low risk by the NRS2002, NUTRIC, and mNUTRIC.

Clinical outcomes	NRS 2002			NUTRIC			mNUTRIC		
	0–4 (*n* = 107)	5–6 (*n* = 127)	*p*-Value	0–2 (*n* = 123)	3–8 (*n* = 111)	*p*-Value	0–2 (*n* = 124)	3–7 (*n* = 110)	*p*-value
28-day mortality (*n*, %)	2 (1.9)	16 (12.6)	0.002	0 (0.0)	18 (16.2)	<0.001	0 (0.0)	18 (16.4)	<0.001
90-day mortality (*n*, %)	4 (3.7)	26 (20.5)	<0.001	0 (0.0)	30 (27.0)	<0.001	1 (0.8)	29 (26.4)	<0.001
ICU length of stay (days)	29 (18–39)	30 (18–47)	0.184	31 (18–40)	29 (18–48)	0.582	31 (18–40)	29 (18–48)	0.681
Surgical intervention (*n*, %)	4 (3.7)	21 (16.5)	0.001	4 (3.3)	21 (18.9)	<0.001	5 (4.0)	20 (18.2)	<0.001
Use of mechanical ventilator (*n*, %)	79 (73.8)	115 (90.6)	<0.001	94 (76.4)	100 (90.1)	<0.001	95 (76.6)	99 (90.0)	<0.001
Renal replacement therapy (*n*, %)	28 (26.2)	55 (43.3)	<0.001	39 (31.7)	44 (39.6)	<0.001	39 (31.5)	44 (40.0)	<0.001
Use of vasoactive agent (*n*, %)	7 (6.5)	29 (22.8)	0.001	10 (8.1)	26 (23.4)	0.001	10 (8.1)	26 (23.6)	0.001
Use of mechanical ventilator >48 h (*n*, %)	6 (5.6)	25 (19.7)	0.002	9 (7.3)	22 (19.8)	0.005	9 (7.3)	22 (20.0)	0.004
Renal replacement therapy >48 h (*n*, %)	20 (18.7)	35 (27.6)	0.111	24 (19.5)	31 (27.9)	0.130	24 (19.4)	31 (28.2)	0.112
Use of vasoactive agent >48 h (*n*, %)	4 (3.7)	18 (14.2)	0.006	5 (4.1)	17 (15.3)	0.003	5 (4.0)	17 (15.5)	0.003
MODS (*n*, %)	13 (12.2)	62 (48.8)	<0.001	20 (16.3)	55 (49.6)	<0.001	21 (16.9)	54 (49.1)	<0.001

### Energy intake and mortality risk analyses

**Figure 3:**
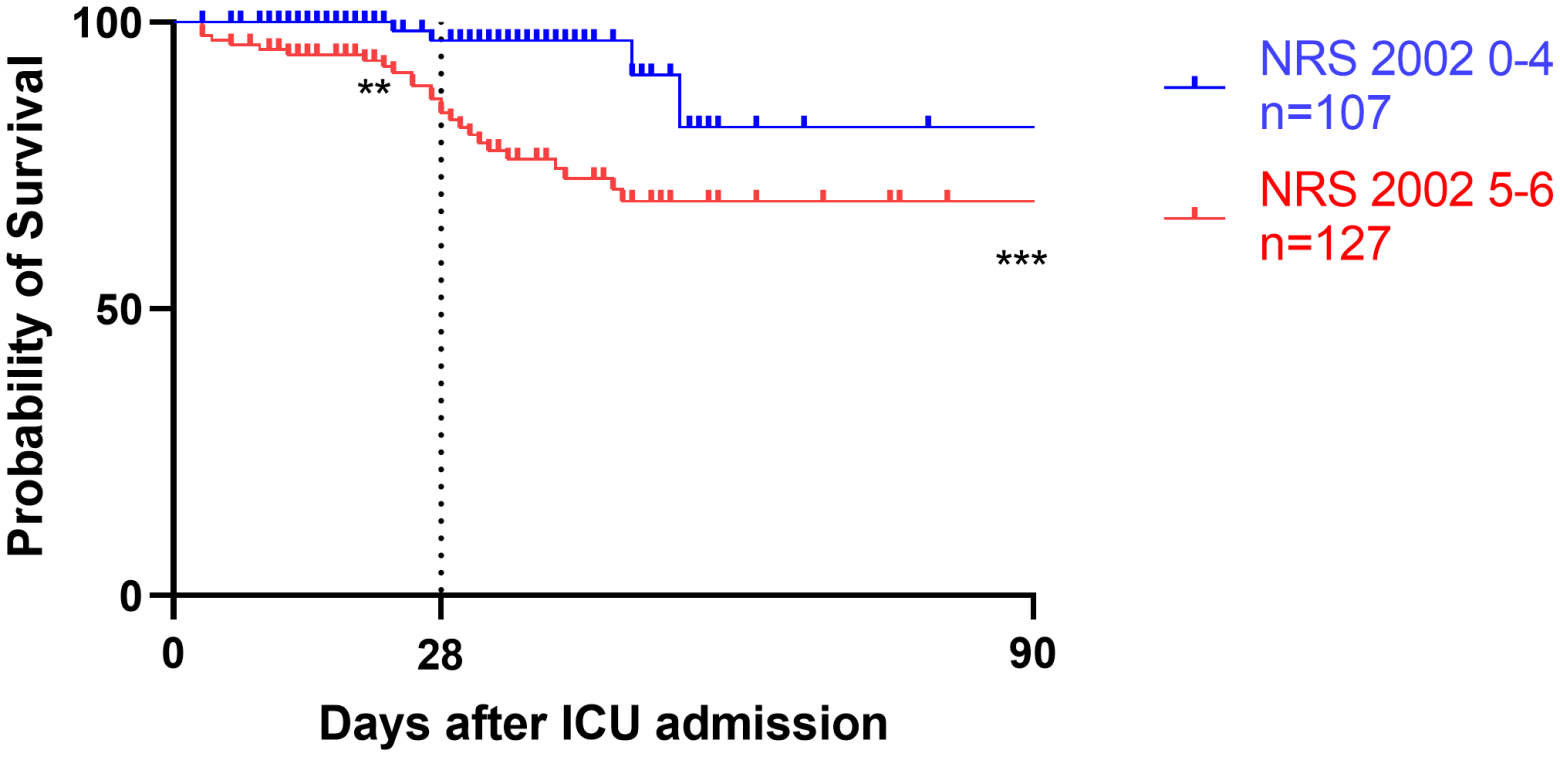
Kaplan–Meier survival analyses depending on baseline scores of low NRS 2002 0-4 (blue line, *n* = 107) versus high NRS 2002 5-6 (red line, *n* = 127); **p < 0.05, ***p < 0.001.

**Figure 4:**
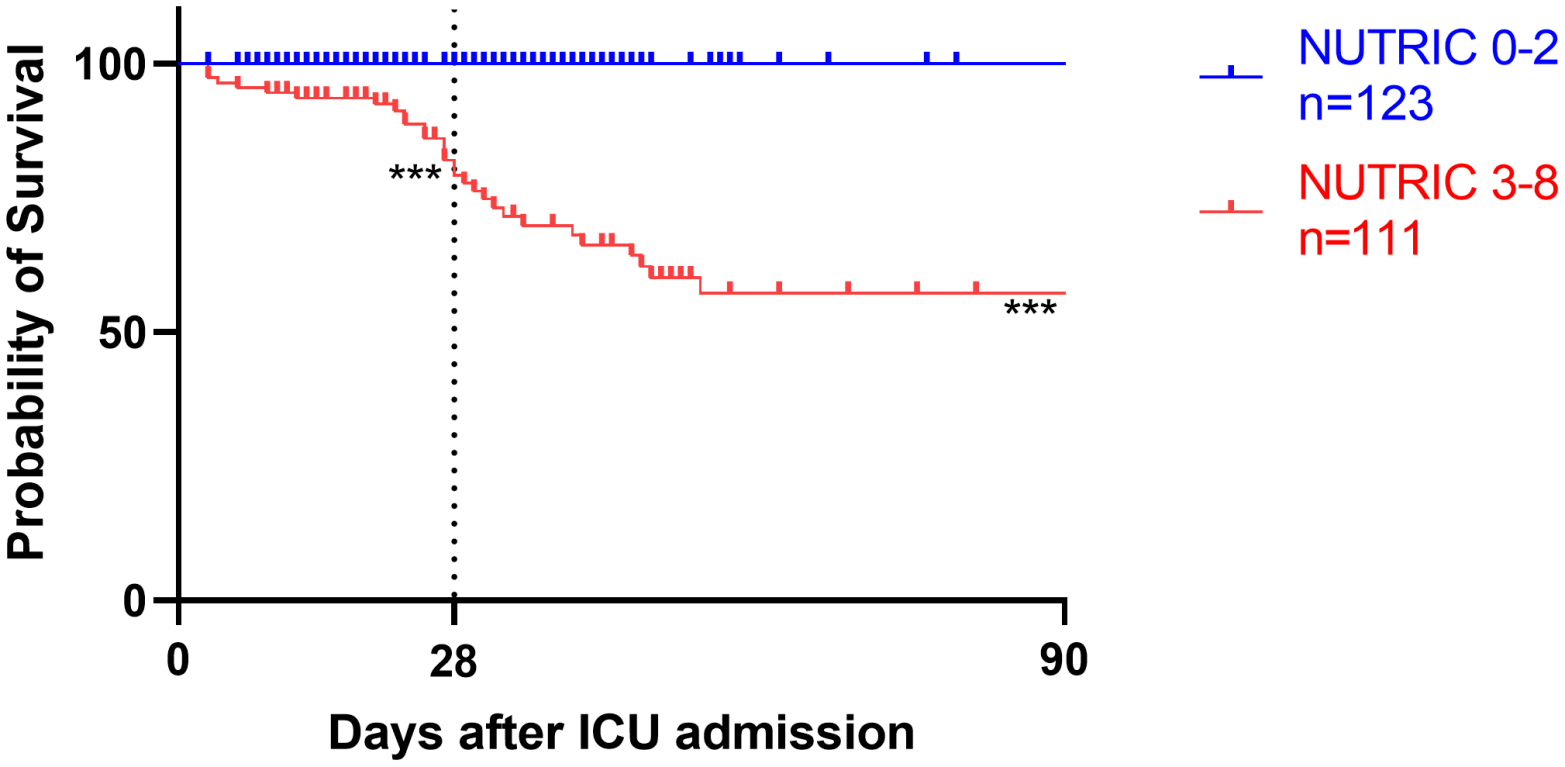
Kaplan–Meier survival analyses depending on baseline scores of low NUTRIC 0-2 (blue line, *n* = 123) versus high NUTRIC 3-8 (red line, *n* = 111); ***p < 0.001.

**Figure 5:**
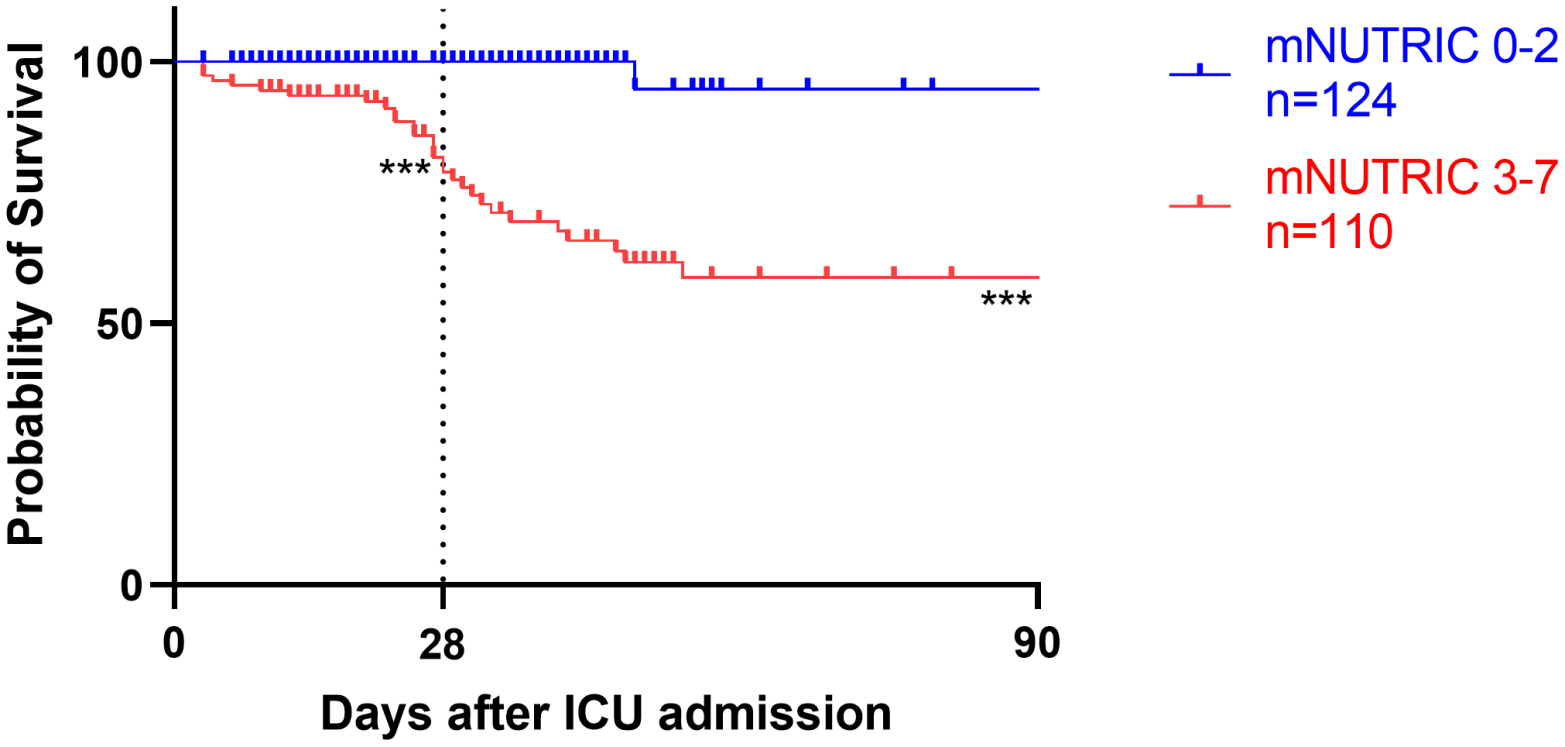
Kaplan–Meier survival analyses depending on baseline scores of low mNUTRIC 0-2 (blue line, *n* = 124) versus high mNUTRIC 3-7 (red line, *n* = 110); ***p < 0.001.

Survival analyses by Kaplan–Meier (Figures 3–5) showed significant differences depending on different scoring levels. By day 28, a total of 18 (7.6%) patients had died. Of these, 16 patients were categorized as high nutritional risk according to the NRS 2002, while all 18 patients were identified as high nutritional risk according to the NUTRIC and mNUTRIC scores and the cut-off values calculated in the previous part of this study. The Cox proportional hazards regression analysis of factors associated with mortality are shown in Table 4. The SAP patients with higher NRS 2002 (HR = 2.889, 95% CI: 1.278–6.528, *p* = 0.011), NUTRIC (HR = 1.691, 95% CI: 1.331–2.148, *p* < 0.001), and mNUTRIC (HR = 1.689, 95% CI: 1.292–2.207, *p* < 0.001) scores had a higher risk of short-term mortality.

**Table 4:** Cox proportional hazards regression model for mortalities.

Screening tools	Unadjusted			Adjusted*		
	HR	95% CI	*p*-Value	HR	95% CI	*p*-Value
28-day mortality						
NRS 2002	3.026	1.469–6.234	0.003	2.889	1.278–6.528	0.011
NUTRIC	1.799	1.436–2.254	<0.001	1.691	1.331–2.148	<0.001
mNUTRIC	1.792	1.393–2.305	<0.001	1.689	1.292–2.207	<0.001
90-day mortality						
NRS 2002	2.605	1.464–4.635	0.001	2.461	1.286–4.713	0.007
NUTRIC	1.852	1.541–2.224	<0.001	1.747	1.441–2.118	<0.001
mNUTRIC	1.788	1.460–2.190	<0.001	1.683	1.359–2.083	<0.001

* Hazard ratio (95% CI) and *p*-value calculated with Cox proportional hazards model with adjustment for baseline value of PCT.

Similar results were also revealed in the long-term mortality. In total, 30 (12.8%) patients had died by day 90. The NRS 2002 (HR = 2.461, 95% CI: 1.286–4.713, *p* = 0.007) failed to identify 4 of these, and the mNUTRIC (HR = 1.683, 95% CI: 1.359–2.083, *p* < 0.001) failed to identify 1 of these, whereas the NUTRIC (HR = 1.747, 95% CI: 1.441–2.118, *p* < 0.001) correctly categorized all patients. The results of the subgroup analyses are reported in Supplementary Figure S3 (28-day mortality) and Supplementary Figure S4 (90-day mortality). The effects of the NRS 2002, NUTRIC, and mNUTRIC were consistent across all subgroups.

**Figure 6:**
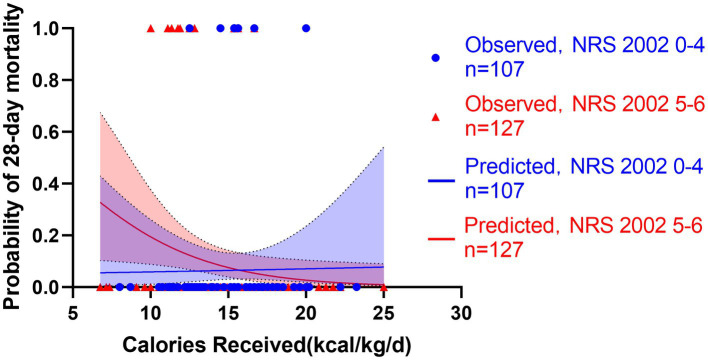
Predicted probability of 28-day mortality versus calories received by low NRS 2002 0-4 (blue line, *n* = 107) versus high NRS 2002 5-6 (red line, *n* = 127). Blue cycles represent the low NRS 2002 cases while red triangles represent the high NRS 2002 cases. Test for interactions were assessed by a logistic model including the NRS 2002, the energy provision and their product (interaction). The NRS 2002 was found unable to modify the relationship between energy provision during first week and 28-day mortality (*p* = 0.166).

**Figure 7:**
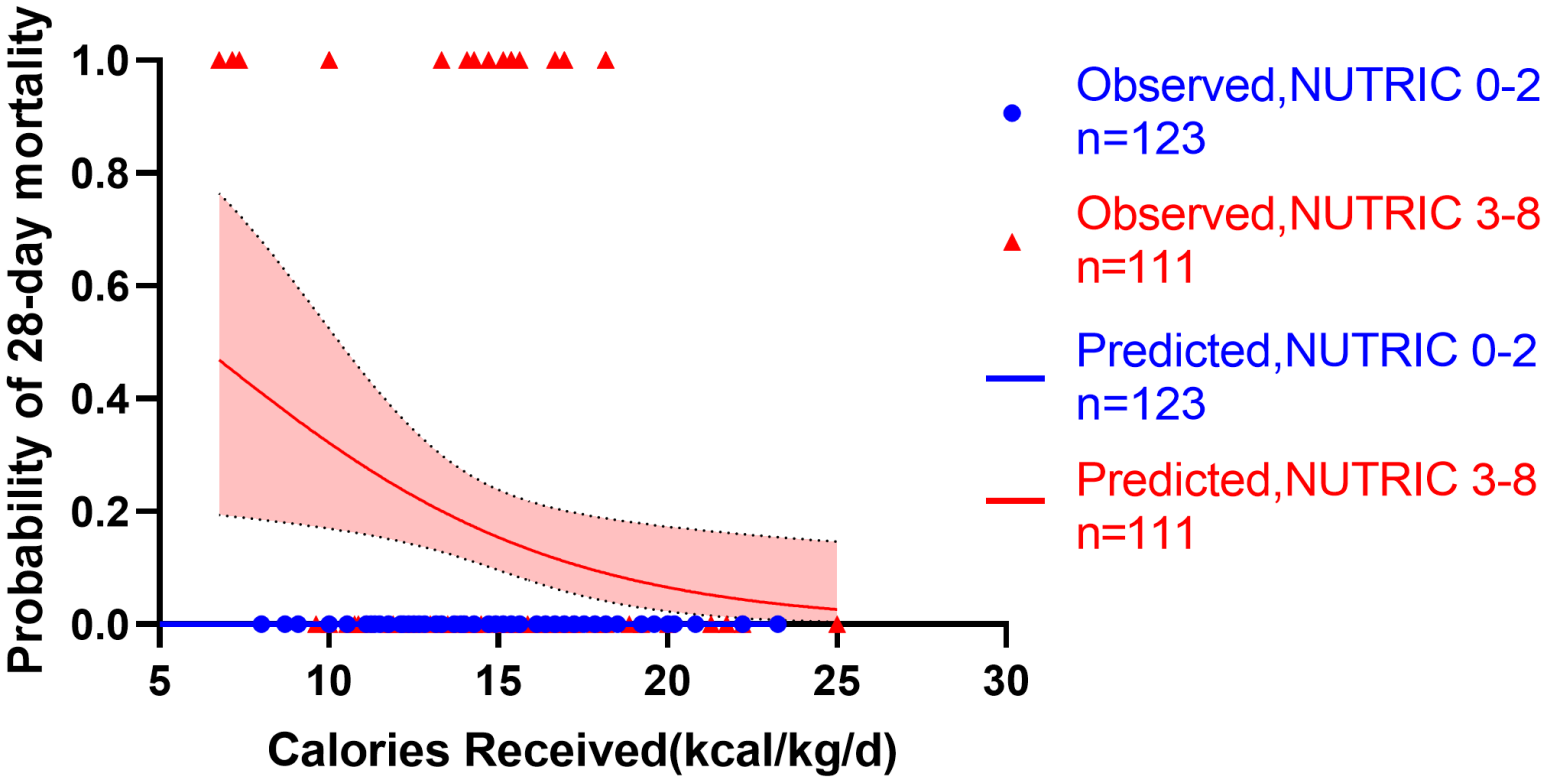
Predicted probability of 28-day mortality versus calories received by low NUTRIC 0-2 (blue line, *n* = 123) versus high NUTRIC 3-8 (red line, *n* = 111). Blue cycles represent the low NUTRIC score cases while red triangles represent the high NUTRIC score cases. Test for interactions were assessed by a logistic model including the NUTRIC score, the energy provision and their product (interaction). The test for interaction confirmed the association between energy provision and mortality is significantly modified by the NUTRIC score (test for interaction *p* < 0.001).

**Figure 8:**
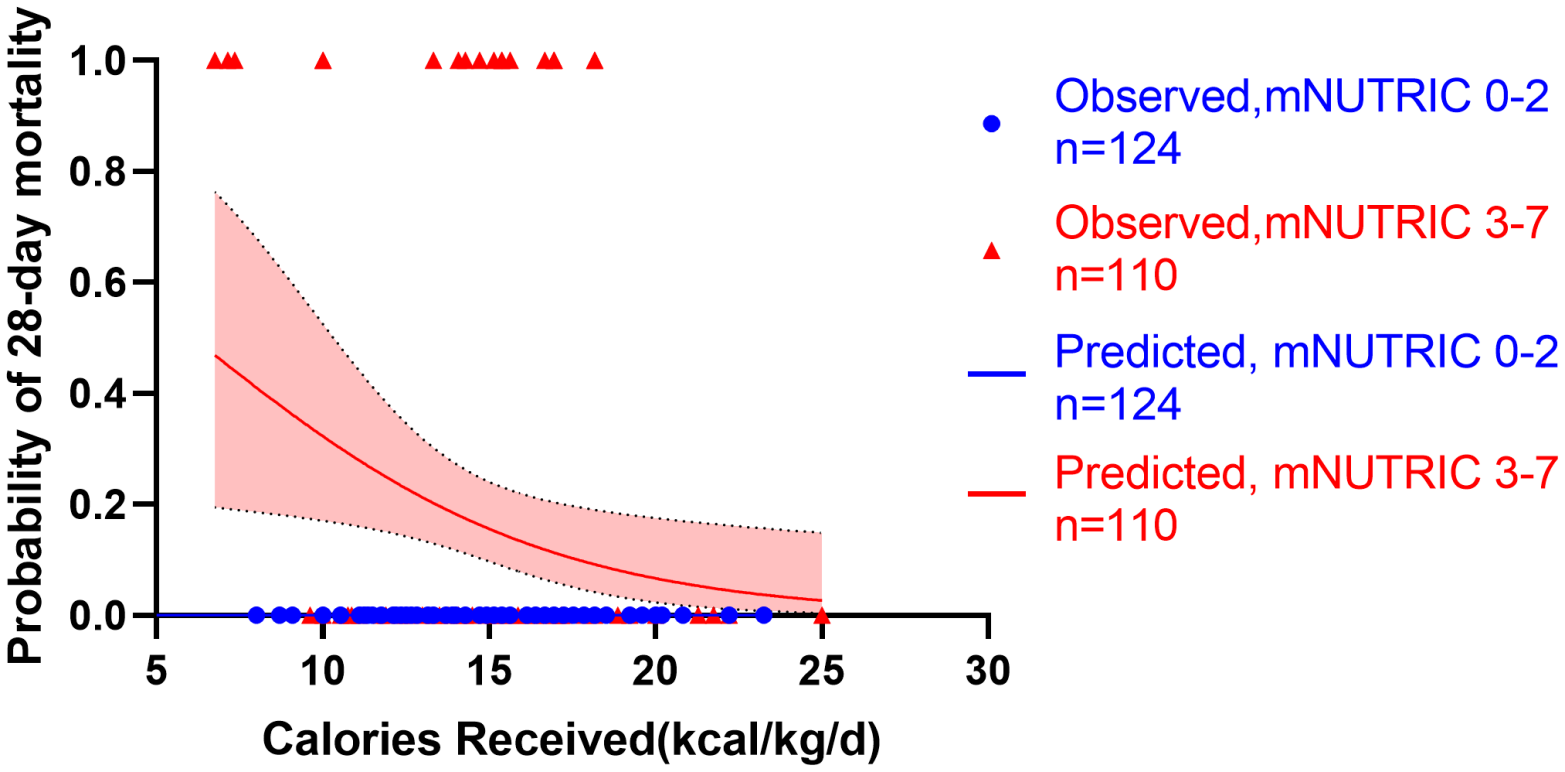
Predicted probability of 28-day mortality versus calories received by low mNUTRIC 0-2 (blue line, *n* = 124) versus high mNUTRIC 3-7 (red line, *n* = 110). Blue cycles represent the low NUTRIC score cases while red triangles represent the high NUTRIC score cases. Test for interactions were assessed by a logistic model including the NUTRIC score, the energy provision and their product (interaction). The test for interaction confirmed the association between energy provision and mortality is significantly modified by the mNUTRIC score (test for interaction *p* < 0.001).

All patients started enteral nutrition by nasogastric or nasojejunal feeding within 72 h after admission. During the first week after admission to ICU, the calories received were 15.4 ± 3.2 kcal/kg/day, on average. The average protein intake was 0.7 ± 0.2 g/day. If the target of calorie target is set as 25–30 kcal/kg/day, in accordance with current guidelines, only 130 (56%) patients received more than 60% of caloric adequacy. The median energy provision was 61.5 with an interquartile range from 53.1 to 68.8. Energy provision was not correlated with the NRS 2002, NUTRIC score or mNUTRIC score. Mortality generally decreased with increasing energy provision, and the Hosmere-Lemeshow goodness of fit test showed that the calibration of the model was statistically ideal (*p* = 0.386). Using these validation data, the logistic model estimated odds of mortality were multiplied by 0.822 (95% CI, 0.682–0.956, *p* = 0.015) for every 1 kcal/kg/day increase on the energy provision. Separate models confirmed the results in high nutritional risk group (high NRS 2002 group, OR = 0.808, 95% CI: 0.701–0.963, *p* = 0.013; high NUTRIC score group, OR = 0.826, 95% CI: 0.702–0.972, *p* = 0.021; and high mNUTRIC score group, OR = 827, 95% CI: 0.703–0.973, *p* = 0.022) while patients categorized as low nutritional risk did not show the benefits of higher energy provision rate (low NRS 2002 group: OR = 1.172, 95% CI: 0.712–1.928, *p* = 0.533; Models were not applicable for no patients died in low NUTRIC or low mNUTRIC group within 28 days). The test for interaction confirmed the association between energy provision and mortality is significantly modified by the NUTRIC score (test for interaction *p* < 0.001) and the mNUTRIC score (test for interaction *p* < 0.001) while the interaction was not modified by the NRS 2002(test for interaction *p* = 0.166). Figures 6–8 demonstrate that increased calorie intake during the first week is associated with increased short-term survival in patients categorized as high nutritional risk by the NUTRIC, or mNUTRIC. No statistical difference was found in the calories received during the first week after admission in different nutritional risk groups. The detailed results were shown in Supplementary Table S1.

## Discussion

Unfortunately, no treatments were proven effective to suppress the powerful cascade of inflammatory factors associated with SAP (17). With this limitation, the current treatment method of SAP is primarily supportive and nutrition support is considered a major method in treating SAP patients. Nutritional risk assessment of SAP patients is an important element for outcome prediction. Many possible features can lead to malnutrition in SAP patients. Many possible features can lead to malnutrition in this population. In this study, considering that two peaks (short-term and long-term) of mortality are observed for SAP patients, we chose to investigate the prognostic accuracy of the NRS 2002, NUTRIC, and mNUTRIC in predicting the 28- and 90-day mortality of SAP patients (18–21). The NUTRIC demonstrated the highest prediction value among the three scoring systems. A similar prognostic accuracy was found for the mNUTRIC. In the absence of IL-6, the mNUTRIC can equally predict the clinical outcomes of SAP patients.

In SAP patients, early death usually occurs as a result of systemic inflammatory response syndrome (SIRS) and MODS (22). SIRS is often caused by the release of various cytokines in the first 2 weeks. Shinzeki et al. (20) reported that early death accounted for 22% (5/23) of all deaths in their study, and we observed a similar figure in our study, wherein 7 (23%) patients died during the first 2 weeks (Supplementary Figure S3) and were well identified by the NUTRIC and mNUTRIC. Acute underfeeding is a possible consequence, and it can lead to immunosuppression and to inflammatory response impairment, which may occur in SAP patients and cause early death. Furthermore, in clinical practice, determining an appropriate target for nutrient supplementation of patients at high risk of malnutrition is crucial. Similar with the previous study, identifying patients with high mNTURIC scores and supporting them with adequate nutrition during an ICU stay would be useful in improving clinical outcomes such as 90-day mortality (23). Due to the nature of the retrospective study, nutrition therapy after day 7 was not included into the analysis, due to the high heterogeneity in the nutrition intake methods. Some patients started oral food intake, and thus, the calorie intake after day 7 was not counted. Hence, we only investigated the effect of nutrition therapy during the first week after admission. In our study, the relationship between nutrition therapy in the first week and short-term mortality was revealed, showing a lower mortality with a higher calorie intake in patients at high nutritional risk. On the other hand, severe disease can also cause acute gastrointestinal injury and a decrease in calorie intake. The organs that most commonly fail in acute pancreatitis include those linked with respiratory, renal, and circulatory failure, while few studies have focused on gastrointestinal failure. Studies have shown that gastrointestinal dysfunction and failure could be an important determinant of outcome in critically ill patients, including acute pancreatitis. Sun et al. (24) suggested that gastrointestinal failure is an accurate predictor of SAP prognosis. Other studies have revealed that gastrointestinal symptoms are frequent in patients in the ICU (25, 26). A total of 62% of patients exhibited at least one gastrointestinal symptom for at least 1 day (27). There is also increasing evidence that the development of gastrointestinal problems is related to a poor outcome in critically ill patients (28). The NUTRIC and mNUTRIC do not include gastrointestinal symptoms, which could thus be a source of bias for a specific group of patients. Regardless, acute underfeeding should be considered as a major complication in SAP.

In the analyses of long-term mortality, the NUTRIC and mNUTRIC also showed better prognostic value than the NRS 2002. As a result, we determined different cut-off values for the NUTRIC and mNUTRIC than a previous study in identifying critically ill SAP patients at a high nutritional risk. According to Youden’s index, we found a cut-off value of ≥3 for both NUTRIC and mNUTRIC to be more appropriate for predicting short-term and long-term mortality. Heyland et al. and Rahman et al. utilized ≥5 (mNUTRIC) and ≥6 (NUTRIC) as cut-off values in critically ill patients when the NUTRIC and mNUTRIC were first introduced (13, 14). De Vries et al. (29) found the best discriminative ability with a mNUTRIC cut-off >4 for 28-day mortality in mechanically ventilated patients. Mayr et al. (30) determined a cut-off value of ≥6 to predict 90-day mortality and a cut-off value of ≥7 to predict 28-day mortality in cirrhotic patients (for both NUTRIC and mNUTRIC). In contrast, Jeong et al. (31) found a cut-off value ≥6 for the mNUTRIC in predicting 28-day mortality. Different cut-off values have thus been found when investigating patients suffering from different diseases. A lower cut-off value of the NUTRIC and mNUTRIC in SAP patients was found in our study in comparison to other studies focusing on different populations. This difference could result from the catabolic nature of SAP and differences in the characteristics of specific diseases. The classic cut-off values cannot well identify SAP patients at nutritional risk, especially using the NRS 2002. All patients from our study were classified as at nutritional risk by utilizing the classic NRS 2002 cut-off value ≥3.

The severity of acute pancreatitis is defined by the presence or absence of organ failure, local complications, or both (1). Local complications or the occurrence of single-organ failure in SAP patients may only result in mild systemic symptoms at the early stage of AP, which could lead to a lower score of SOFA and APACHE II at admission. Lower Glasgow coma scores were observed in SAP patients compared with other critically ill patients upon admission to the ICU. A severe complication of SAP is acute gastrointestinal injury, which cannot be well stratified by the NUTRIC and mNUTRIC. Acute gastrointestinal injury is often underestimated while it could be lethal in SAP patients. All these reasons could result in a lower cut-off value of the NUTRIC and mNUTRIC in SAP patients. Also, APACHE II score <8 at admission may be a predictive factor for the risk of death in 90 days among patients categorized as high nutritional risk by NUTRIC and mNUTRIC (interaction *p* < 0.05). Patients at nutritional risk with lower APACHE II score appeared to have more co-morbidity and delayed longer before admission to ICU than those without. The results for analysis of secondary outcomes were similar to those of the primary outcomes. Only the prediction ability of MODS was rather accurate, with an AUC ≥ 0.75, although the NUTRIC score still showed a better prediction ability for most secondary outcomes. Explanation for the shortcomings of the NUTRIC score is that mortality was the only consideration in the study design when it was first developed (13). Some experts claim that mortality is not the only outcome that should be assessed when determining the efficacy of a nutritional intervention, considering the numerous factors influencing ICU mortality (16). Long-term functional tests might better reflect the benefit of a nutritional intervention and should be included in the screening tools (32). The present results of our study underline the need for further studies utilizing individualized nutritional risk assessment tools based on the NRS 2002, NUTRIC, mNUTRIC or other scoring systems.

Although this study included a reasonable number of patients with SAP, and the results of this study were conclusive with statistical significance, this study has several limitations. The first is that it is a retrospective single-center study. The 28- and 90-day mortalities are defined as the primary outcomes, as there is no gold standard to judge the fitness and accuracy of a nutritional risk screening tool. The study focuses on the assessment of the NRS2002, NUTRIC, and mNUTRIC scores, which were obtained at the time of admission to ICU, whereas no further evaluation was conducted during the course of the disease in the ICU. Although the NUTRIC and mNUTRIC show good prognostic value for 90-day mortality, many other factors should be taken into consideration. Moreover, no interventions occurred during the study. Nutrition therapy after day 7 was not included in the analyses due to the heterogeneity in nutrition intake methods thereafter. The effects of the nutritional therapy on the outcomes of patients with high nutritional risk were thus not fully assessed. Further prospective interventional studies focusing on nutrition therapy based on the NRS2002, NUTRIC, or mNUTRIC are needed to support the findings.

## Conclusion

In conclusion, the NUTRIC and mNUTRIC demonstrated a prognostic advantage comparison with the NRS 2002 in predicting SAP patients at high nutritional risk. Moreover, both NUTRIC and mNUTRIC scores can adequately identify SAP patients at high nutritional risk.

## Data availability statement

The original contributions presented in the study are included in the article/Supplementary material, further inquiries can be directed to the corresponding author.

## Ethics statement

The studies involving human participants were reviewed and approved by Ruijin Hospital Ethics Committee, Shanghai Jiaotong University School of Medicine. Written informed consent for participation was not required for this study in accordance with the national legislation and the institutional requirements.

## Author contributions

DC and BZ drafted and wrote the manuscript. DC, BZ, LW, and YQ collected the data. WG, XB, EC, and FJ analyzed the data. DC, BZ, and JH designed the study. EM, HS, and JH revised the manuscript and supervised the work. All authors contributed to the article and approved the submitted version.

## Conflict of interest

The authors declare that the research was conducted in the absence of any commercial or financial relationships that could be construed as a potential conflict of interest.

## Publisher’s note

All claims expressed in this article are solely those of the authors and do not necessarily represent those of their affiliated organizations, or those of the publisher, the editors and the reviewers. Any product that may be evaluated in this article, or claim that may be made by its manufacturer, is not guaranteed or endorsed by the publisher.
