# Supplementary material for: Prognostic performance of the NRS2002, NUTRIC, and modified NUTRIC to identify high nutritional risk in severe acute pancreatitis patients
Source: Front Nutr. 2023 Mar 2;10:1101555. doi: 10.3389/fnut.2023.1101555 (PMC10017740; doi:10.3389/fnut.2023.1101555)
Supplement: Supplementary file 1 [file Table_1.DOCX]

Supplementary Material

Table S1. Comparison of patient characteristics in SAP patients categ1orized as high nutritional risk and low risk by the NRS2002, NUTRIC and mNUTRIC

| **Variables** | **NRS 2002** | | | **NUTRIC** | | | **mNUTRIC** | | |
| --- | --- | --- | --- | --- | --- | --- | --- | --- | --- |
|  | 0-4 (n=107) | 5-6 (n=127) | *P-*value | 0-2 (n=123) | 3-8 (n=111) | *P-*value | 0-2 (n=124) | 3-7 (n=110) | *P-*value |
| Demographics |  |  |  |  |  |  |  |  |  |
| Age (years) | 44 (34-55) | 52 (40-66) | <0.001 | 41 (33-49) | 57 (48-68) | <0.001 | 41 (33-49) | 57 (48-68) | <0.001 |
| Sex (male,%) | 73 (68.2) | 83 (65.4) | 0.678 | 80 (65.0) | 76 (68.5) | 0.579 | 80 (64.5) | 76 (69.1) | 0.459 |
| BMI (kg/m2) | 24.0 ± 3.8 | 24.2 ± 3.9 | 0.817 | 24.1 ± 4.2 | 24.1 ± 3.5 | 0.930 | 24.1 ± 4.2 | 24.1 ± 3.5 | 0.928 |
| APACHE II | 5 (2-7) | 12 (10-16) | <0.001 | 6 (3-9) | 12 (8-17) | <0.001 | 6 (3-9) | 12 (8-17) | <0.001 |
| SOFA | 3 (1-4) | 6 (4-8) | <0.001 | 3 (2-4) | 6 (4-9) | <0.001 | 3 (2-4) | 6 (4-9) | <0.001 |
| CT severity index at admission | 6 (4-6) | 6 (4-6) | 0.934 | 6 (4-6) | 6 (4-6) | 0.821 | 6 (4-6) | 6 (4-6) | 0.775 |
| MAP at admission (mmHg) | 100 (92-110) | 102 (92-111) | 0.975 | 100 (92-110) | 102 (92-111) | 0.844 | 100 (91-110) | 102 (92-111) | 0.714 |
| Calories received within first week (kcal/kg/d) | 15.6 ± 2.8 | 15.3 ± 3.4 | 0.538 | 15.5 ± 3.0 | 15.4 ± 3.3 | 0.782 | 15.5 ± 3.0 | 15.4 ± 3.4 | 0.784 |
| Etiology |  |  | 0.018 |  |  | 0.001 |  |  | 0.001 |
| Biliary (n,%) | 34 (31.8) | 57 (44.9) |  | 37 (30.1) | 54 (48.6) |  | 38 (30.6) | 53 (48.2) |  |
| Alcoholic (n,%) | 36 (33.6) | 26 (20.5) |  | 40 (32.5) | 22 (19.8) |  | 40 (32.3) | 22 (20.0) |  |
| Hypertriglyceridemia (n,%) | 32 (29.9) | 30 (23.6) |  | 40 (32.5) | 22 (19.8) |  | 40 (32.3) | 22 (20.0) |  |
| Other (n,%) | 5 (4.7) | 14 (11.0) |  | 6 (4.9) | 13 (11.7) |  | 6 (4.8) | 13 (11.8) |  |
| Laboratory test |  |  |  |  |  |  |  |  |  |
| PCT at admission (ng/mL) | 0.7 (0.3-1.7) | 2.3 (0.6-11.5) | <0.001 | 0.7 (0.3-2.3) | 2.2 (0.7-11.8) | <0.001 | 0.7 (0.3-2.2) | 2.3 (0.7-11.9) | <0.001 |
| CRP at admission (mg/L) | 161 (75-227) | 192 (109-268) | 0.024 | 181 (105-244) | 176 (70-262) | 0.490 | 180 (105-242) | 178 (70-262) | 0.500 |
| White-cell count at admission (/mm^3^) | 12045 (9025-16975) | 13500 (10290-18290) | 0.133 | 12915 (9178-17345) | 13100 (10550-16880) | 0.761 | 13000 (9180-17390) | 13060 (10360-16775) | 0.917 |
| Serum creatinine at admission (mg/dL) | 0.7 (0.6-0.9) | 1.0 (0.7-2.0) | <0.001 | 0.7 (0.6-0.9) | 1.0 (0.7-1.9) | <0.001 | 0.7 (0.6-0.9) | 1.0 (0.7-1.9) | <0.001 |
| Serum amylase at admission (IU/L) | 392 (148-785) | 636 (282-1265) | 0.005 | 410 (155-738) | 819 (278-1435) | 0.001 | 417 (158-744) | 807 (274-1403) | 0.001 |
