# Supplementary material for: Prognostic performance of the NRS2002, NUTRIC, and modified NUTRIC to identify high nutritional risk in severe acute pancreatitis patients
Source: Front Nutr. 2023 Mar 2;10:1101555. doi: 10.3389/fnut.2023.1101555 (PMC10017740; doi:10.3389/fnut.2023.1101555)
Supplement: Supplementary file 2 [file Data_Sheet_1.PDF]

Supplementary Material

Supplementary Figures

(A)

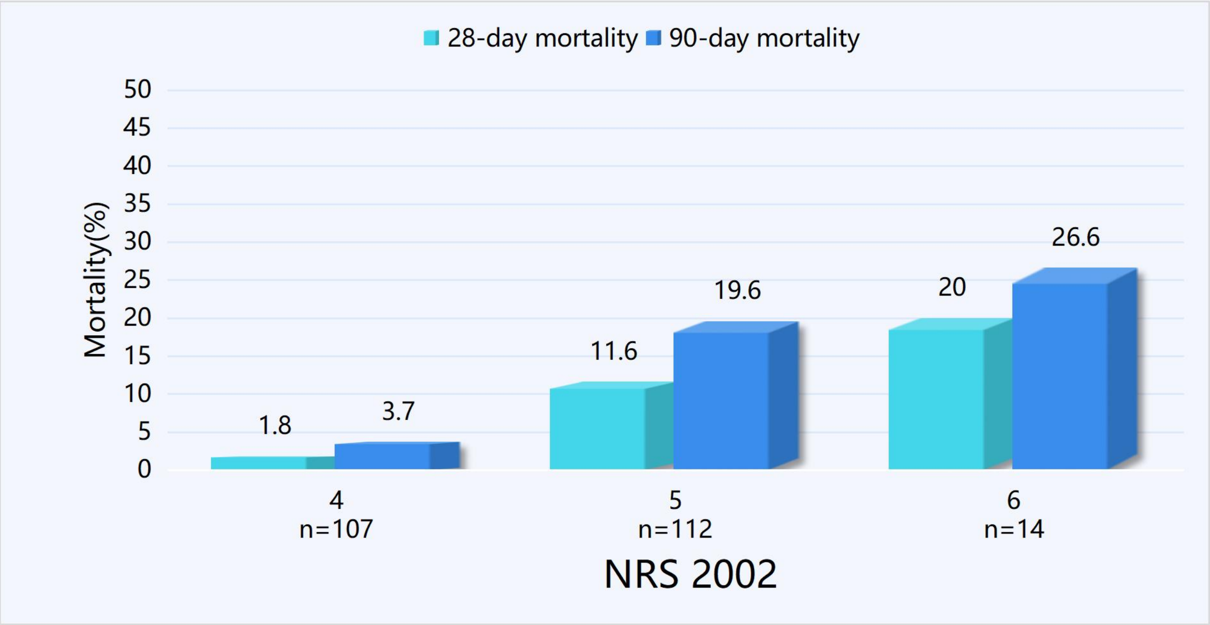

(B)

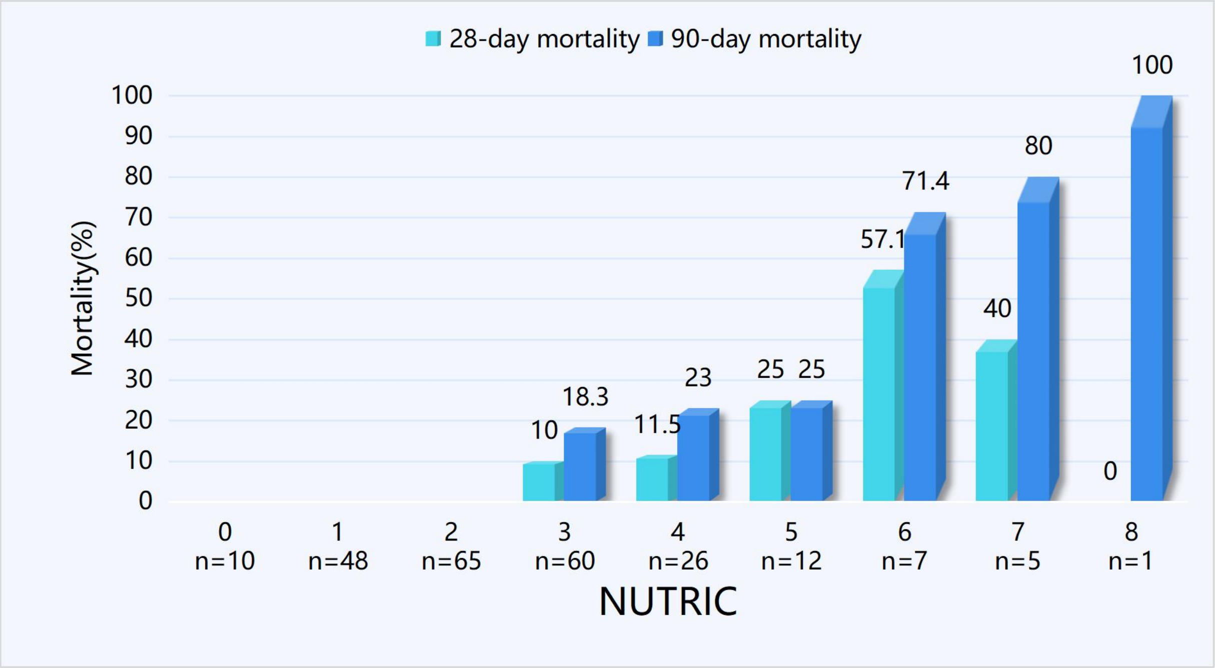

(C)

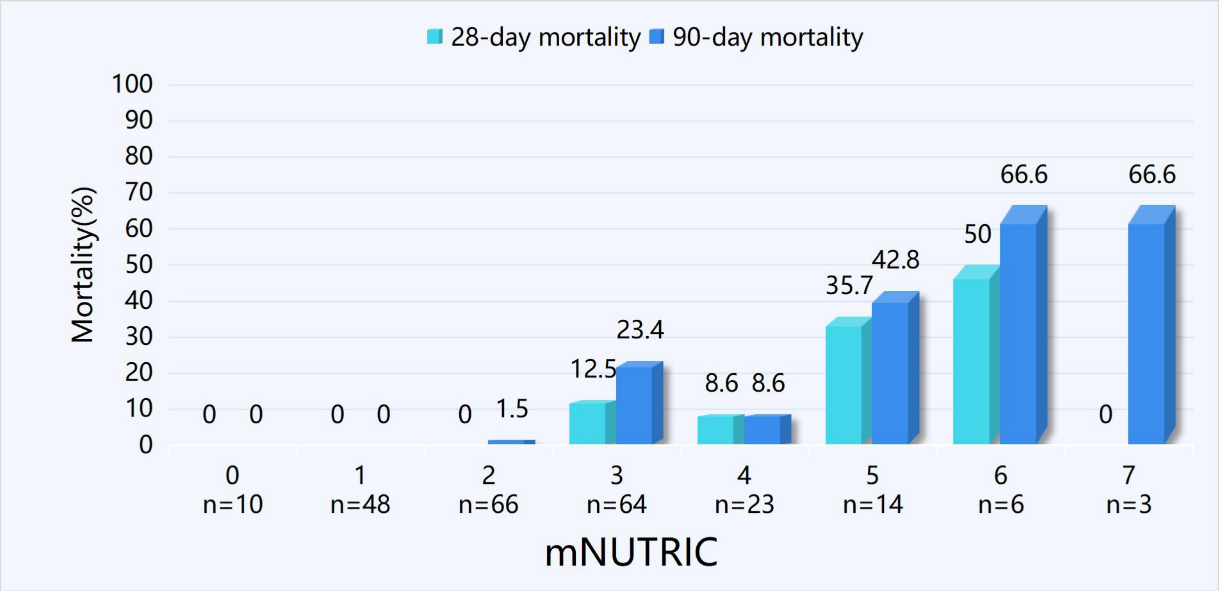

Figure S1. 28-day and 90-day mortality after admission categorized by (A)NRS 2002,(B)NUTRIC and (C)mNUTRIC.

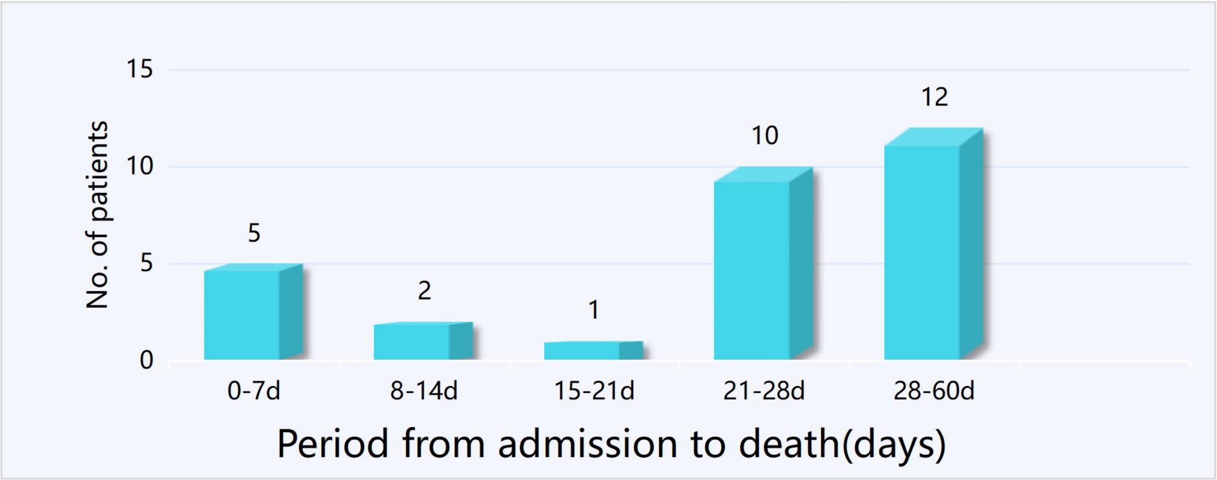

Figure S2. The period from admission to death.

(A)

Subgroups	No. of Events/Total (%) NRS 2002 0-4	5-6	Hazard ratio (95%CI)	*P* for interaction
Sex				
Male	2/73(2.7)	12/83(14.5)	2.928(1.043-8.226)	0.844
Female	0/34(0.0)	4/44(9.1)	3.367(0.796-14.252)	
Age(years)				
>55	1/30(3.3)	8/56(14.3)	2.136(0.808-5.648)	0.532
≤55	1/77(1.3)	8/71(11.3)	4.418(0.514-37.958)	
APACHE II				
≥8	0/20(0.0)	14/115(12.2)	2.431(0.785-7.526)	0.348
<8	2/87(2.3)	2/12(16.7)	5.201(0.688-39.139)	
WBC(/mm^3)				
>16000	0/32(0.0)	4/42(9.5)	3.094(0.560-17.085)	0.977
≤16000	2/75(2.7)	12/85(14.1)	2.858(1.135-7.195)	
CT severity index				
>6	0/14(0.0)	6/16(37.5)	4.858(1.073-21.990)	0.328
≤6	2/93(2.2)	10/111(9.1)	2.052(0.771-5.456)	
CRP(mg/L)				
>150	0/56(0.0)	10/88(11.4)	2.170(0.759-6.202)	0.327
≤150	2/51(3.9)	6/39(15.4)	4.813(1.408-16.451)	
SCr(mg/dL)				
≥1.8	0/3(0.0)	10/37(27.0)	3.776(0.814-17.510)	0.541
<1.8	2/104(1.9)	6/90(6.7)	2.254(0.799-6.355)	
Etiology				
Biliary	1/34(2.9)	9/57(15.8)	2.245(0.913-8.226)	0.584
Other	1/73(1.4)	7/70(14.5)	3.599(0.757-17.108)	

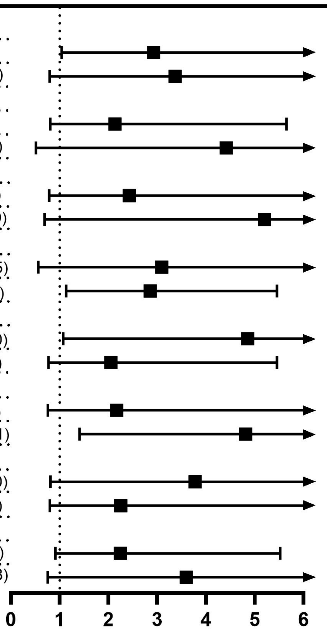

(B)

Subgroups	No. of Events/Total (%) NUTRIC 0-2	3-8	Hazard ratio (95%CI)	*P* for interaction
Sex				
Male	0/80(0.0)	14/76(18.4)	1.772(1.362-2.306)	0.155
Female	0/43(0.0)	4/35(11.4)	1.478(0.918-2.378)	
Age(years)				
>55	0/22(0.0)	9/64(14.1)	1.542(1.071-2.220)	0.109
≤55	0/101(0.0)	9/47(19.2)	2.184(1.389-3.432)	
APACHE II				
≥8	0/45(0.0)	14/90(15.6)	1.682(1.264-2.240)	0.008
<8	0/78(0.0)	4/21(19.1)	7.590(1.453-39.637)	
WBC(/mm^3)				
>16000	0/42(0.0)	4/32(9.5)	3.176(1.287-7.836)	0.609
≤16000	0/81(0.0)	14/79(17.7)	1.671(1.287-2.168)	
CT severity index				
>6	0/15(0.0)	6/15(40.0)	1.227(0.798-1.888)	0.186
≤6	0/108(0.0)	12/96(12.5)	1.984(1.438-2.737)	
CRP(mg/L)				
>150	0/77(0.0)	10/67(14.9)	1.939(1.404-2.679)	0.170
≤150	0/46(0.0)	8/44(18.2)	1.544(0.991-2.406)	
SCr(mg/dL)				
≥1.8	0/9(0.0)	10/31(32.3)	1.846(1.190-2.863)	0.407
<1.8	0/114(0.0)	8/80(10.0)	1.789(1.152-2.776)	
Etiology				
Biliary	0/37(0.0)	10/54(18.5)	1.997(1.374-2.901)	0.279
Other	0/86(0.0)	8/57(14.0)	1.631(1.031-2.581)	

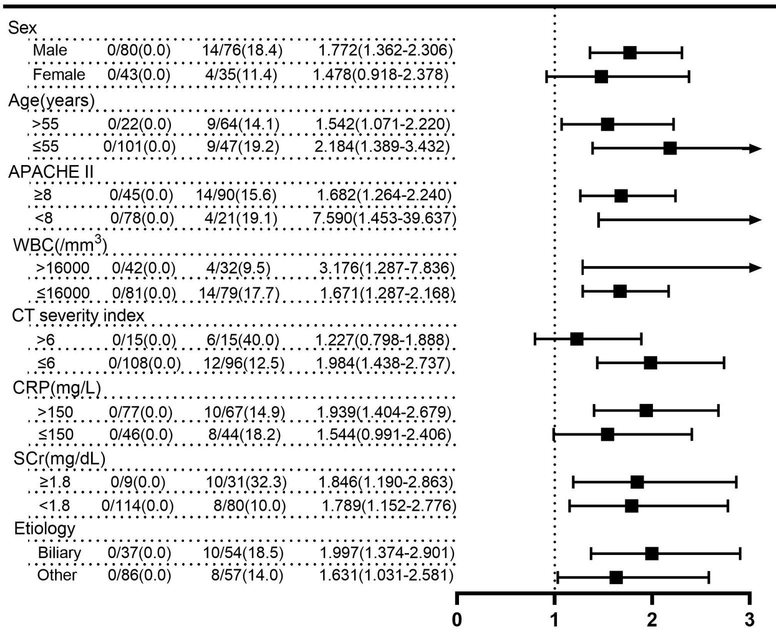

(C)

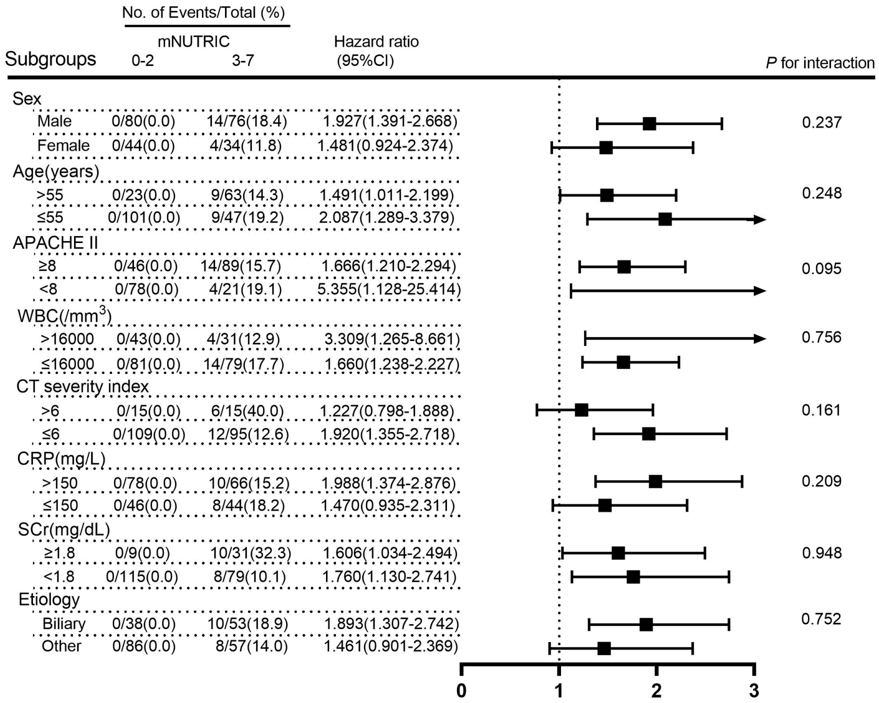

Figure S3. Risk of death in 28 days in the prespecified subgroups among patients. (A) NRS 2002, (B) NUTRIC, and (C) mNUTRIC.

(A)

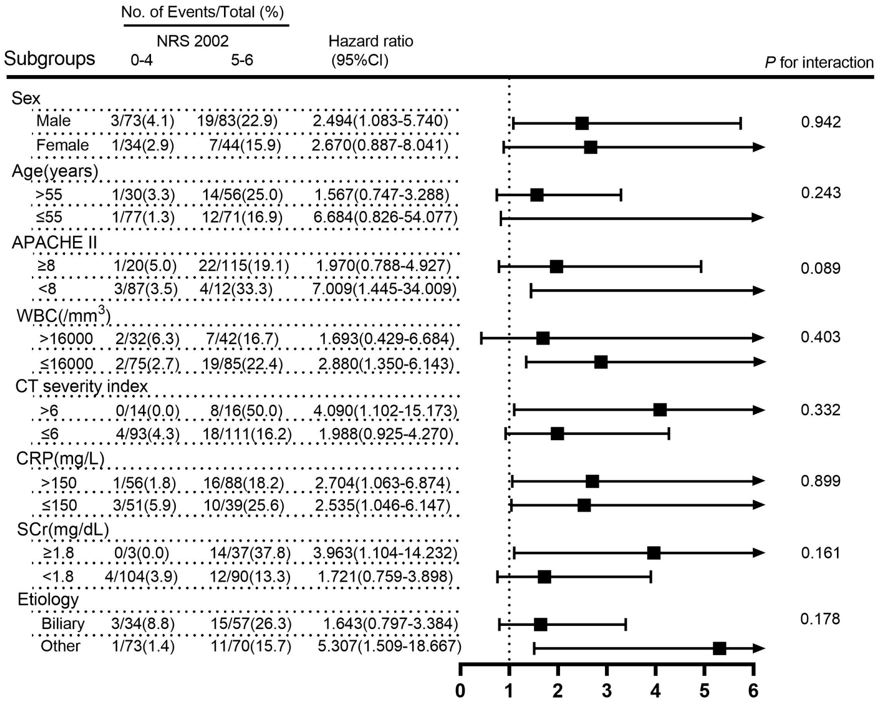
No. of Events/Total (%)
NRS 2002
Subgroups
0-4
5-6
Hazard ratio
(95%CI)
P for interaction
Sex
Male 3/73(4.1) 19/83(22.9) 2.494(1.083-5.740) 0.942
Female 1/34(2.9) 7/44(15.9) 2.670(0.887-8.041)
Age(years)
>55 1/30(3.3) 14/56(25.0) 1.567(0.747-3.288) 0.243
≤55 1/77(1.3) 12/71(16.9) 6.684(0.826-54.077)
APACHE II
≥8 1/20(5.0) 22/115(19.1) 1.970(0.788-4.927) 0.089
<8 3/87(3.5) 4/12(33.3) 7.009(1.445-34.009)
WBC(/mm3)
>16000 2/32(6.3) 7/42(16.7) 1.693(0.429-6.684) 0.403
≤16000 2/75(2.7) 19/85(22.4) 2.880(1.350-6.143)
CT severity index
>6 0/14(0.0) 8/16(50.0) 4.090(1.102-15.173) 0.332
≤6 4/93(4.3) 18/111(16.2) 1.988(0.925-4.270)
CRP(mg/L)
>150 1/56(1.8) 16/88(18.2) 2.704(1.063-6.874) 0.899
≤150 3/51(5.9) 10/39(25.6) 2.535(1.046-6.147)
SCr(mg/dL)
≥1.8 0/3(0.0) 14/37(37.8) 3.963(1.104-14.232) 0.161
<1.8 4/104(3.9) 12/90(13.3) 1.721(0.759-3.898)
Etiology
Biliary 3/34(8.8) 15/57(26.3) 1.643(0.797-3.384) 0.178
Other 1/73(1.4) 11/70(15.7) 5.307(1.509-18.667)
0 1 2 3 4 5 6

(B)

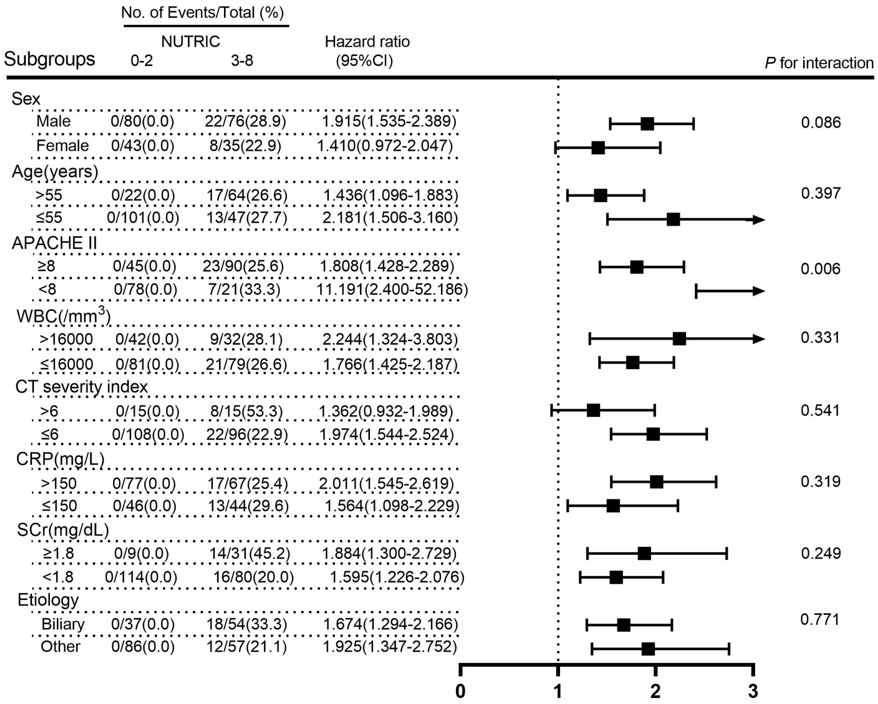
No. of Events/Total (%)
NUTRIC
Subgroups
0-2
3-8
Hazard ratio
(95%CI)
P for interaction
Sex
Male 0/80(0.0) 22/76(28.9) 1.915(1.535-2.389) 0.086
Female 0/43(0.0) 8/35(22.9) 1.410(0.972-2.047)
Age(years)
>55 0/22(0.0) 17/64(26.6) 1.436(1.096-1.883) 0.397
≤55 0/101(0.0) 13/47(27.7) 2.181(1.506-3.160)
APACHE II
≥8 0/45(0.0) 23/90(25.6) 1.808(1.428-2.289) 0.006
<8 0/78(0.0) 7/21(33.3) 11.191(2.400-52.186)
WBC(/mm3)
>16000 0/42(0.0) 9/32(28.1) 2.244(1.324-3.803) 0.331
≤16000 0/81(0.0) 21/79(26.6) 1.766(1.425-2.187)
CT severity index
>6 0/15(0.0) 8/15(53.3) 1.362(0.932-1.989) 0.541
≤6 0/108(0.0) 22/96(22.9) 1.974(1.544-2.524)
CRP(mg/L)
>150 0/77(0.0) 17/67(25.4) 2.011(1.545-2.619) 0.319
≤150 0/46(0.0) 13/44(29.6) 1.564(1.098-2.229)
SCr(mg/dL)
≥1.8 0/9(0.0) 14/31(45.2) 1.884(1.300-2.729) 0.249
<1.8 0/114(0.0) 16/80(20.0) 1.595(1.226-2.076)
Etiology
Biliary 0/37(0.0) 18/54(33.3) 1.674(1.294-2.166) 0.771
Other 0/86(0.0) 12/57(21.1) 1.925(1.347-2.752)
0 1 2 3

(C)

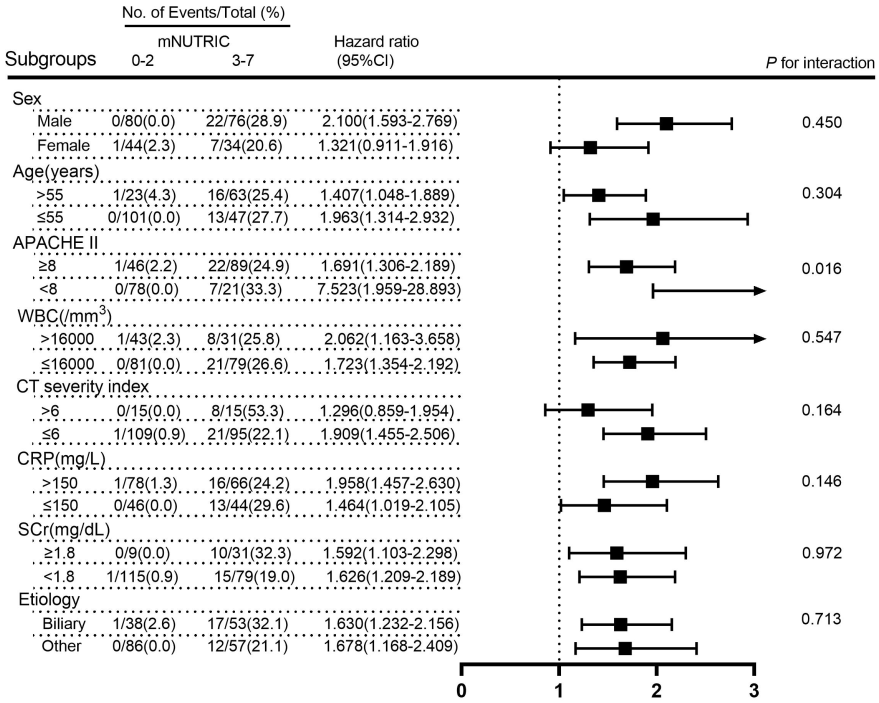

Figure S4. Risk of death in 90 days in the prespecified subgroups among patients. (A) NRS 2002, (B) NUTRIC, and (C) mNUTRIC.
